# Editorial: Women in Aging Research

**DOI:** 10.3389/fragi.2022.913488

**Published:** 2022-05-20

**Authors:** Consuelo Borrás, Laura Haynes, Jianhua Zhang

**Affiliations:** ^1^ Freshage Research Group, Department of Physiology, Faculty of Medicine, Centro de Investigación Biomédica en Red Fragilidad y Envejecimiento Saludable-Instituto de Salud Carlos III (CIBERFES-ISCIII), INCLIVA, University of Valencia, Valencia, Spain; ^2^ UConn Center on Aging, University of Connecticut School of Medicine, Farmington, CT, United States; ^3^ Department of Pathology, University of Alabama-Birmingham, Birmingham, AL, United States

**Keywords:** urinary tract, neural control, intestinal, lung, heart failure, sex

In this Women in Aging Research Topic which was launched and completed in honor of International Women’s Day, we celebrate women researchers in the Aging field. Furthermore, we highlight research perspectives on sex differences in aging and age-related diseases. This is important, as until recently, diseases and aging in females were not extensively studied in preclinical research.

In an insightful and comprehensive review, Kobak et al. evaluated animal models that investigate age and sex differences in heart failure with preserved ejection fraction (HFpEF). HFpEF is a devastating age-dependent disease without effective treatment. Sex-dependent mechanisms have been proposed. With multi-organ manifestation, systemic features, and multiple observed risk factors, animal models that explore accelerated aging, hypertension, metabolic stress, multi-hit, and large animal models were studied. This review summarized observations using these animal models, their limitations, and their utility in enhancing our understanding of HFpEF, which will no doubt serve as an essential guide and reference for future studies. Notably, L-NAME (nitric oxide synthase inhibitor) and high-fat diet (HFD) double hit mouse model exhibit sex different phenotypes. However, it does not seem that any single animal model can recapitulate all the HFpEF phenotypes in humans. Furthermore, there is still an unmet need to determine sex differences, especially in aged animal models. Noteworthy to mention that Kobak et al. was trained with Dr. Rabinovitch at the University of Washington studying the effect of rapamycin on the old heart and now established herself as an assistant professor at Oklahoma medical research foundation with a special research interest in HFpEF, and the effects of caloric restriction and targeting mTOR on the aging heart.

Sex differences have also been reviewed by a group led by Kehmeier and Walker from the Aging and Vascular Physiology Laboratory at the University of Oregon, focusing on large artery stiffness, which underlies cerebrovascular dysfunction and Alzheimer’s disease. This occurs more rapidly in females between ∼55 and 75 years of age when compared to males and has a stronger association with mortality in females. Mechanisms of sex differences in large artery stiffness may involve sex hormones, collagen content, nitric oxide bioavailability, blood flow, and pressure pulsatility, cerebrovascular endothelial dysfunction.

Mishraet al. is a professor at U Connecticut Health, with a primary interest in the basic biology of aging, with a special focus on female longevity. In the review published by Mishraet al., research on INDY (I am Not Dead Yet, a fly gene homologous to the mammalian SLC13A5 which encodes a plasma membrane citrate transporter) was evaluated across several species, including flies, worms, mice, rats, non-human primates and humans. Major findings include the connection to energy metabolism and cell proliferation, and the effects of INDY disruption on bone, intestinal stem cells, heart, and brain in mammals. Species-specific effects of INDY loss of function have also been noted, which might be explained by differences in biochemical and biophysical properties of the protein in different organisms. Yet further studies of sex differences and disruption of INDY function later in life rather than from embryonic development are essential to guide the development and testing of small molecules that target INDY to treat diseases including cancer and metabolic diseases and promote healthy aging.

Reduced capacity of protein degradation is one of the important hallmarks of aging that may lead to misfolded protein accumulation and progressive loss of function in organ systems. In this research topic, Demirel-Yalciner et al. review the importance of the endoplasmic reticulum (ER) systems to recognize these altered proteins. On the other hand, miRNAs are involved in shaping the ER stress, while miRNA expression is also regulated by ER stress, so she discusses how ER stress–miRNA relationship is involved in aging and different diseases such as cancer, cardiovascular, metabolic, and neurodegenerative diseases. Demirel-Yalciner et al. is a professor at Marmara University (Istambul, Turkey) and she was President of the Society for Free Radical Research_Europe around 10 years ago. Her research focuses on endoplasmic reticulum stress, redox signaling, and cell death in metabolism-related diseases.

Rocha is an assistant professor at the University of Coimbra (Portugal) and her studies are centered on the biology of nitric oxide (NO) in the gut through a recently described non-enzymatic pathway: the nitrate-nitrite-nitric oxide pathway. Indeed, in this research topic, she discusses the role of this pathway in pre-clinical and clinical data regarding the impact of dietary nitrate on the elderly. Nitric oxide is a small hydrophobic gas, that interacts with molecular targets ensuring physiological functions such as vasodilation, innate immune response, and neuromodulation. There are several endogenous sources of NO, but it can be obtained also from the diet in high quantities. Therefore, the nitrate intake in the diet may contribute so much to the beneficial effects of NO in aging and age-related diseases such as those related to the cardiovascular, nervous, and musculoskeletal systems.

Urinary symptoms increase with aging and women are more affected than men. Hardy reviews the role of neural control over micturition, which is significantly impacted by aging processes. Aging increases the probability of incomplete bladder emptying and decreases sensitivity to volume, as aged individuals are, in general, less reliable in transmitting accurate information on bladder content through the central nervous system. Treatments usually involve side effects that complicate their use in the elderly and comorbidities often complicate surgical procedures as well, so adequate interventions with impact on neuronal signaling need to be explored. Hardy is a postdoctoral fellow at UConn Center on Aging (Farmington, United States) with high experience in urinary physiology and microsurgery in animal models.

McMahan et al. group from the Department of Surgery at the University of Colorado, Anschutz Medical Campus contributed an original research article on how aging impacts *Streptococcus pneumoniae* infection. Their findings demonstrated that infections in older animals resulted in increased lung neutrophilia along with potentially pathogenic alterations in commensal gut bacteria and highlight potential mechanistic targets contributing to the increased morbidity and mortality observed with aging.

We hope that this Research Topic provides readers with the breath of aging research areas and new insights contributed by women researchers, stimulates new ideas, and encourages further research efforts that advance the field.

